# Glycolate is a Novel Marker of Vitamin B_2_ Deficiency Involved in Gut Microbe Metabolism in Mice

**DOI:** 10.3390/nu12030736

**Published:** 2020-03-11

**Authors:** Takashi Uebanso, Ayumi Yoshimoto, Shinta Aizawa, Maya Nakamura, Rumiko Masuda, Takaaki Shimohata, Kazuaki Mawatari, Akira Takahashi

**Affiliations:** 1 Department of Preventive Environment and Nutrition, Institute of Biomedical Sciences, Tokushima University Graduate School, 3-18-15, Kuramoto, Tokushima 770-8503, Japan; sea.by15.koko@gmail.com (A.Y.); c201831026@tokushima-u.ac.jp (S.A.); ztyet396@yahoo.co.jp (M.N.); masuda.rumiko@tokushima-u.ac.jp (R.M.); shimohata@tokushima-u.ac.jp (T.S.); mawatari@tokushima-u.ac.jp (K.M.); akiratak@tokushima-u.ac.jp (A.T.); 2 Department of Nutrition, Faculty of Health Science, Hyogo University, 2301, Hiraokacho, Shinzaike, Kakogawa 675-0195, Japan

**Keywords:** glycolate, vitamin B_2_, gut microbe, metabolome

## Abstract

Microbes in the human gut play a role in the production of bioactive compounds, including some vitamins. Although several studies attempted to identify definitive markers for certain vitamin deficiencies, the role of gut microbiota in these deficiencies is unclear. To investigate the role of gut microbiota in deficiencies of four vitamins, B_2_, B_6_, folate, and B_12_, we conducted a comprehensive analysis of metabolites in mice treated and untreated with antibiotics. We identified glycolate (GA) as a novel marker of vitamin B_2_ (VB2) deficiency, and show that gut microbiota sense dietary VB2 deficiency and accumulate GA in response. The plasma GA concentration responded to reduced VB2 supply from both the gut microbiota and the diet. These results suggest that GA is a novel marker that can be used to assess whether or not the net supply of VB2 from dietary sources and gut microbiota is sufficient. We also found that gut microbiota can provide short-term compensation for host VB2 deficiency when dietary VB2 is withheld.

## 1. Introduction

Recent studies have highlighted the presence of trillions of microbes in the human gut and their role in host health and disease [1,2,3,4]. Dietary nutrients and other environmental factors are key components that regulate gut microbe populations, and these bacteria produce several metabolites that are fundamental for biological processes in the host, including absorption, metabolism, and storage of ingested nutrients [5,6,7,8]. Studies in both mice and humans have shown the effects that gut microbiota have on host metabolism. For example, fermentation of polysaccharides by gut microbiota produces a range of short-chain fatty acids (SCFAs), including acetate, butyrate, and propionate [6]. SCFAs are substrates for energy production (lipogenesis and gluconeogenesis) and also affect various cellular processes (e.g., proliferation, differentiation, and modulation of gene expression) [9]. Gut bacteria in the ileum modulate the transformation of primary and conjugated bile acids into secondary bile acids [6]. Primary bile acids are important for absorption of dietary fat and fat-soluble vitamins from entero-hepatic circulation. Bile acids also function as signaling molecules and regulate cellular processes, such as those mediated by the farnesoid X receptor (FXR) and the G-protein coupled receptor TGR5 [10,11,12].

Gut microbes have long been known to contribute to supplies of bioactive compounds, particularly vitamins [13,14]. Administration of folate-producing *Bifidobacteria* enhances folate status in rats and humans [15,16]. In mice, deficiencies in biotin, also known as vitamin B_7_, induce alopecia that can be exacerbated by overgrowth of the biotin-consuming bacteria *Lactobacillus murinus* [17]. Other B vitamins, such as vitamin B_6_ and B_12_, could regulate bacterial toxicity by interacting with host and gut commensal bacteria, as well as with enteropathogenic bacteria [18,19]. Thus, gut microbiota both produce and use vitamins to provide functional metabolites for the host and other bacteria. 

Vitamins are critical nutrients that support metabolic processes needed to sustain homeostasis in mammals. In particular, one-carbon (1C) metabolism supports multiple physiological processes, wherein 1C is transferred through processes associated with folate and methionine metabolism [20,21]. These processes include purine and thymidylate biosynthesis, amino acid metabolism, epigenetic maintenance, and redox responses. Intracellular folate and methionine metabolism are crucial components of these metabolic pathways. Vitamin B_2_ (riboflavin), B_6_ (pyridoxine, pyridoxal, and pyridoxamine), and B_12_ act as cofactors of 1C metabolism to sustain the folate cycle coupled with the methionine cycle (Supplementary Figure S1). 

Although several attempts have been made to reveal definitive markers of vitamin deficiency, the role of gut microbiota in these deficiencies is unclear. Several methods that combine separation methods with mass spectrometry (MS) were recently developed that allow comprehensive analysis of metabolites, called the metabolome. In the present study, we used MS methods to examine the role of gut microbiota in mice fed a diet deficient in B_2_, B_6_, folate, and B_12_ by evaluating gut luminal content, as well as performing analyses of tissues and the plasma metabolome. We found that GA is a novel marker of VB2 deficiency, and that the gut microbiota can also sense VB2 deficiency. This novel marker responds to reductions in VB2 supply from both the gut microbiota and the diet. Moreover, these results suggest that GA could be valuable to assess the net supply of VB2 from the gut microbiota and the diet.

## 2. Materials and Methods

### 2.1. Animals

Nine-week-old female C57Bl/6J mice were purchased from a local breeding colony (Charles River Japan, Yokohama, Japan) and acclimatized for 1 week prior to use in Experiments 1–7. Mice were housed in cages maintained at a constant temperature (23 °C ± 2 °C) and humidity (65%–75%) with a 12 h light–dark cycle (8:00 a.m. to 20:00 p.m.). For urine collection, mice were placed in metabolic cages for 14 h (18:00 p.m. to 8:00 a.m.). AIN93G was used as the control diet (Oriental Yeast, Osaka Japan). A vitamin B-deficient (VB-) diet, lacking vitamin B_2_, B_6_, B_12_, and folic acid (B_9_); a vitamin B_2_ (VB2)-deficient (VB2-) diet; and a vitamin B_6_ (VB6)-deficient (VB6-) diet were also used. For VB6 or VB2 supplementation, 6 μg/mL pyridoxine hydrochloride (Tokyo Kasei, Tokyo, Japan) or flavin mononucleotide sodium salt (Wako, Osaka, Japan) in distilled water was used to adjust the dietary intake of these nutrients in AIN93G-fed mice. To disrupt the gut microbiota, a mixture of four antibiotics (Ab): penicillin V (Tokyo Kasei), ampicillin (Sigma, St. Louis MO, USA), metronidazole (Tokyo Kasei), each at 150 mg/mouse/day; and vancomycin (WAKO) at 75 mg/mouse/day were administered. Animals were allowed food and water *ad libitum* throughout the experimental period. At the end of the experimental period, all mice were euthanized at noon for collection of blood, cecum, luminal content, feces, and liver tissue. The University of Tokushima Animal Use Committee approved the study (T14010 and T28-84), and mice were maintained according to the National Institutes of Health guidelines for care and use of laboratory animals.

### 2.2. Vitamin Measurement

Plasma, blood, and urine VB6 and/or VB2 levels were measured using VitaFast Vitamin B_6_ and B_2_ kits (Azmax, Chiba, Japan) according to the manufacturer’s instructions.

### 2.3. Glycolate Oxidase Activity Assay

Hepatic glycolate oxidase (GO) activity was measured as reported [22,23]. Briefly, hepatic proteins were extracted in 10 volumes of assay buffer (My Bioscience M1358243218). Protein sample solutions were homogenized, sonicated, and centrifuged for 15 min at 12,000× *g* and 4 °C. Protein concentrations of the supernatants were measured by the bicinchoninic acid method (Thermo Fisher Scientific, Bremen, Germany) and used as samples. GO converts GA to glyoxylate with concomitant H_2_O_2_ production that promotes oxidation of o-Dianisidine (Tokyo Kasei) by horse radish peroxidase (Tokyo Kasei). The absorbance at 415 nm was measured by spectrophotometry and the relative activity was calculated with respect to the amount of protein in the sample.

### 2.4. Metabolome Analysis by Capillary Electrophoresis Electrospray Ionization Time-of-Flight Mass Spectrometry

All samples were prepared according to methods described by Human Metabolome Technologies, Inc. (HMT) (HMT, Tsuruoka, Japan) and in previous reports [24,25,26]. Briefly, the cecum luminal content, stool, cecum, liver, urine, and plasma were immediately frozen in liquid nitrogen and stored at −80 °C until metabolite extraction. Samples were weighed and completely homogenized in ice-cold methanol containing internal standards. All samples were analyzed by capillary electrophoresis electrospray ionization time-of-flight mass spectrometry (CE-TOFMS) on an Agilent CE system combined with a TOFMS (Agilent Technologies, Palo Alto, CA, USA), as reported previously [27,28]. Each metabolite was identified and quantified based on the peak information, including *m/z*, migration time, and peak area. 

### 2.5. Statistical Analyses

All values are expressed as mean ± S.E. The significance of differences between two groups was assessed using an unpaired two-tailed *t* test. Analysis of variance (ANOVA) or the Kruskal–Wallis test was used to make comparisons between more than two groups. When a significant difference was found by the ANOVA or Kruskal–Wallis test, post hoc analyses were performed using the Tukey–Kramer protected least significant difference test. Two-way ANOVA was used to determine the effect of two factors and their interaction. Repeated measures ANOVA was used to estimate time-dependent effects. Spearman’s rank correlation coefficient was used to calculate correlation coefficients between selected variables. Differences were considered significant at *p* < 0.05. Statistical analyses were performed using Mass profiler Professional (MPP) and Excel-Toukei 2006 (SSRI).

## 3. Results

**Figure 1:**
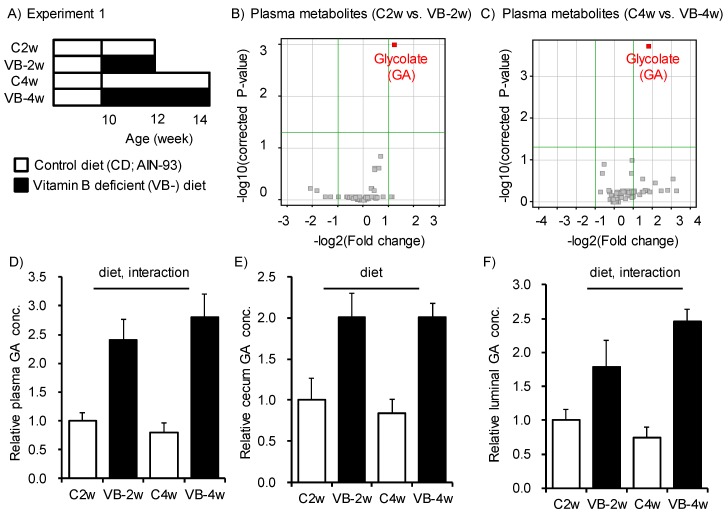
Feeding of diet deficient in vitamin B_2_, B_6_, B_12_, and folic acid induced accumulation of glycolate in plasma, cecum, and luminal content of the cecum. (**A**) Study design for Experiment 1. Mice were fed a control diet (C: AIN93-G) or a diet deficient in vitamin B_2_, B_6_, B_12_, and folic acid (VB-) diet for 2 or 4 weeks. (**B**,**C**) Volcano plots of 77 metabolites in plasma from mice fed either VB- or control diet for (**B**) 2 or (**C**) 4 weeks. The glycolate (GA) concentration changed significantly in both the 2- and 4-week feeding groups. (**D**–**F**) Relative concentration of glycolate in (**D**) plasma, (**E**) cecum, and (**F**) luminal content for the four groups (*n* = 4–5). Two-way ANOVA was used to determine the effect of diet, time, and the interaction of diet and time; *p* < 0.05.

To address how the vitamin B_2_, B_6_, folic acid (B_9_), and B_12_-deficient diet (VB-) alters the plasma metabolome in mice, we conducted a CE-TOFMS analysis to examine specific changes after feeding for 2 and 4 weeks (Figure 1A). We identified 77 metabolites in plasma from a metabolite list provided by HMT. A volcano plot indicated that levels of glycolate (Glycolic acid: GA) were increased in both the 2- and 4-week VB- feeding group (Figure 1B,C), and the relative concentration of the other 76 metabolites did not differ among each group (Supplementary Figure S2). The increase in the GA concentration in plasma was larger for the 4-week feeding group (VB-4w) than the 2-week feeding group (VB-2w, Figure 1D). The luminal content and cecum from the 2- and 4-week VB- diet groups both had higher GA concentrations than the respective control groups (Figure 1E,F). Accumulation of GA in the luminal content and cecum suggests that gut microbiota may contribute to GA metabolism.

**Figure 2:**
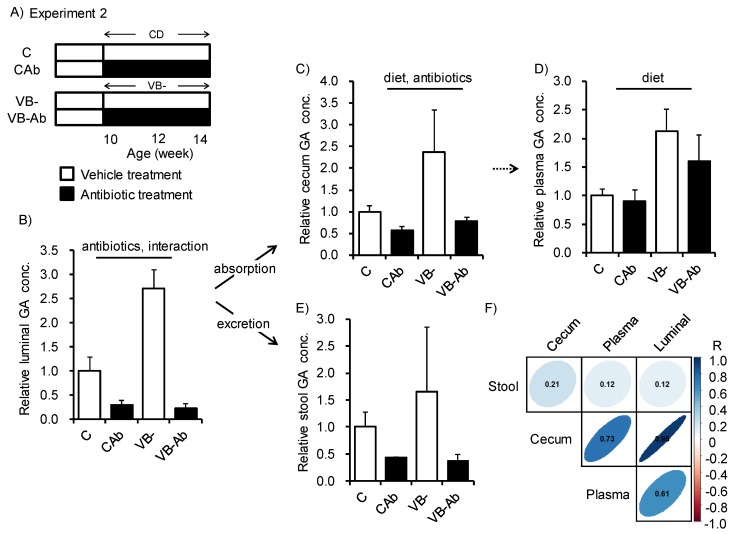
Both the gut microbiota and host accumulated glycolate in mice fed the VB- diet. (**A**) Study design for Experiment 2. Mice were fed the control diet or VB- diet for 4 weeks with or without treatment with four different antibiotics (150 mg/mouse/day penicillin V, ampicillin, metronidazole, and 75 mg/mouse/day vancomycin solved in distilled water). (**B**–**E**) Relative concentration of GA in (**B**) luminal content, (**C**) cecum, (**D**) plasma, and (**E**) stool among the four groups (*n* = 3–4). Two-way ANOVA was used to determine the effect of diet, antibiotics, and the interaction of the two. *p* < 0.05. (**F**) Spearman’s rank correlation coefficient used to describe correlation between luminal GA concentration and that in cecum, plasma, and stool had values of 0.95, 0.61, and 0.12, respectively.

To investigate how the gut microbiota is involved in changes in GA metabolism upon VB- feeding, the gut microbiota was disrupted by delivery of antibiotic (Ab) mixtures for 4 weeks together with VB- diet feeding (Figure 2A). The total numbers of bacteria in feces from the Ab-treated group were significantly reduced (Supplemental Figure S3). Ab-treated mice also exhibited reduced luminal and cecum GA concentrations (Figure 2B,C), whereas the plasma GA concentration was affected only by vitamin B deficiency and not by the antibiotic treatment (Figure 2D). The GA concentration in the stool varied among individuals in the groups (Figure 2E). The correlation coefficients between luminal GA concentration and that of the cecum, plasma, and stool were 0.95, 0.61, and 0.12, respectively (Figure 2F). These results indicated that luminal GA was strongly affected by vitamin B deficiency in intestinal tissues, including the cecum, but was not sufficient to alter plasma GA levels. Moreover, both the gut microbiome and host exhibited similar metabolic changes in response to vitamin B_2_, B_6_, B_12_, and folic acid deficiency.

**Figure 3:**
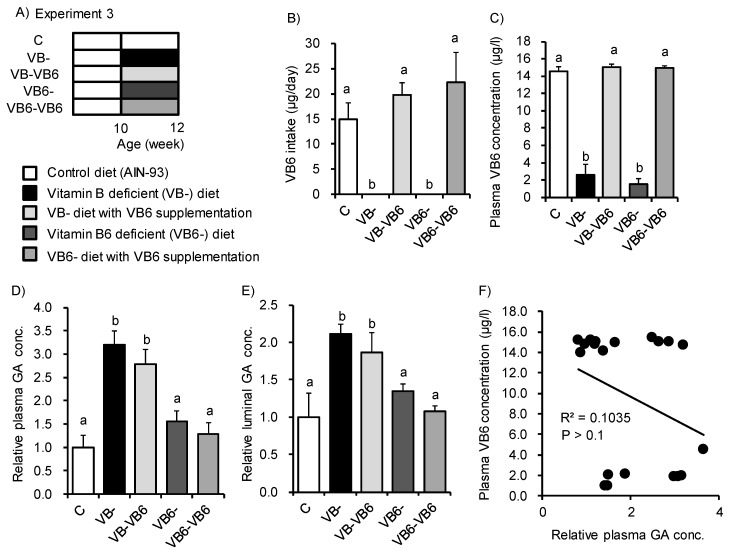
Feeding of a vitamin B_6_-deficient diet did not induce glycolate accumulation in plasma or luminal content. (**A**) Study design for Experiment 3. Mice were fed the control diet, VB- diet, or vitamin B_6_-deficient (VB6-) diet for 2 weeks with or without VB6 supplementation (6 μg/mL pyridoxine hydrochloride solution in distilled water). (**B**) Average daily VB6 intake and (**C**) plasma VB6 concentration for the five groups. (**D**,**E**) Relative concentration of GA in (**D**) plasma and (**E**) luminal content for the five groups. (**F**) Spearman’s rank correlation coefficients between plasma VB6 concentration and plasma GA levels (R^2^ = 0.1035, *p* > 0.1). ANOVA was used to determine the differences in each group (*n* = 4). Different letters indicate *p* < 0.05.

Next, we examined what type of vitamin B deficiency enhanced GA accumulation. Ogawa et al. reported that rats fed the VB6-deficient diet showed increased plasma GA concentrations [29]. VB6 is a cofactor of alanine-glyoxylate aminotransferase1 (AGT1) that catalyzes the transformation of glyoxylate into glycine [30]. When AGT1 is impaired, GA and oxalate are produced as compensatory products by glycolate oxidase (GO) and lactate dehydrogenase, respectively. Here, we used VB6 supplementation to complement VB6 deficiency caused by the VB- diet (Figure 3A). We also used a diet deficient in VB6 alone (VB6-) to investigate the direct effects of VB6 deficiency. Mice fed the VB- diet or VB6- diet for two weeks displayed significant reductions in VB6 intake and plasma VB6 concentration. These decreases were recovered following VB6 supplementation (Figure 3B,C). However, the plasma and luminal GA concentration were not changed by the VB6- diet or by VB6 supplementation (Figure 3D,E). Indeed, the plasma GA concentration did not correlate with the plasma VB6 concentration (R^2^ = 0.1035, *p* > 0.1, Figure 3F). These results indicate that VB6 deficiency does not have a central role in the GA increase seen in mice. 

**Figure 4:**
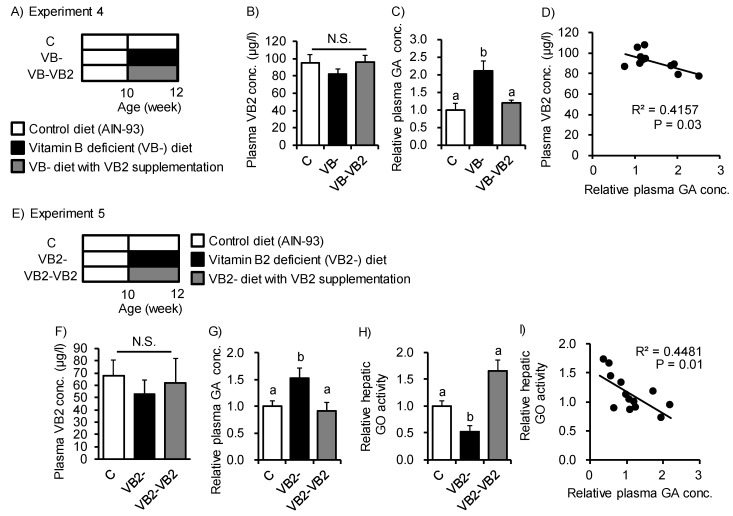
Feeding of a vitamin B_2_-deficient diet induced glycolate accumulation in plasma from mice. (**A**,**E**) Study design for Experiments 4 and 5. Mice were fed a control diet, VB- diet, or VB2-deficient (VB2-) diet for 2 weeks with or without VB2 supplementation (6 μg/mL flavin mononucleotide sodium salt solution in distilled water). (**B**,**F**) Average plasma VB2 concentration in each of the three groups. (**C**,**G**) Relative plasma concentration of GA. (**D**) Spearman’s rank correlation coefficients between plasma VB2 concentration and plasma GA levels (r^2^ = 0.42, *p* = 0.03). (**H**) Relative GA oxidase (GO) activity in the liver. (**I**) Spearman’s rank correlation coefficients between relative hepatic GO activity and plasma GA levels (r^2^ = 0.45, *p* = 0.01). ANOVA was used to determine differences in each group (n = 3–4 in Experiment 4 and n = 4–5 in Experiment 5). Different letters indicate *p* < 0.05 and N.S. represents no significant difference among the groups.

GO metabolizes GA into glyoxylate in the peroxisome. Impairment of GO activity induced elevations in urinary GA excretion in GO-deficient mice [31]. In this context, VB2 acts as a GO cofactor [32]. Therefore, we next examined the effect of VB2 deficiency on GA accumulation induced by the VB- diet (Figure 4A,E). Feeding of the VB- diet or VB2-deficient (VB2-) diet for 2 weeks slightly—but not significantly—reduced plasma VB2 concentrations, even though VB2 intake was reduced (Supplemental Figure S4A,B and Figure 4B,F). Meanwhile, the GA concentration in plasma increased with both VB- and VB2- diet feeding and the levels were restored to those of the control by VB2 supplementation (Figure 4C,G). The plasma GA concentration was significantly correlated with the plasma concentration of VB2 (R^2^ = 0.42, *p* = 0.03; Figure 4D). Accordingly, hepatic GO activity was reduced with VB2 deficiency and recovered by VB2 supplementation (Figure 4H). Hepatic GO activity was negatively correlated with plasma GA levels (Figure 4I). Together, these results suggest that VB2- diet feeding suppresses hepatic GO activity and could promote accumulation and secretion of GA to the bloodstream. 

**Figure 5:**
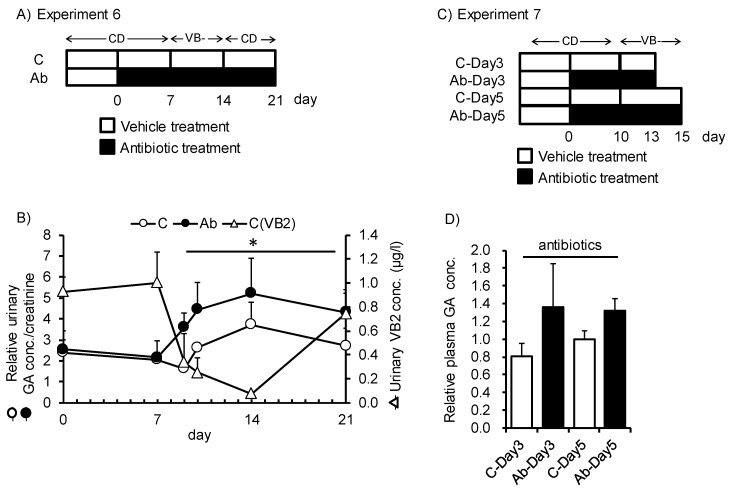
Disruption of gut microbiota following rapid depletion of VB2 in mice fed VB- diet for a short period. (**A**,**C**) Study design for Experiments 6 and 7. Mice were switched from the control diet to the VB- diet after treatment with antibiotics or the vehicle as a control. Urine or plasma samples were obtained over the indicated time period. (**B**) Time-dependent changes in creatinine-adjusted urinary GA concentration (white and black circles indicate the control and antibiotics-treated mice, respectively) or urinary VB2 concentration (white triangle in the control group). (**D**) Relative concentration of GA in plasma at different time points. Repeated measured ANOVA was used to determine the differences in each group (n = 3). * indicates *p* < 0.05. Two-way ANOVA was used to determine the effect of time, antibiotics, and the interaction of the two.

Finally, we investigated the capacity of gut microbiota to be a source of VB2 for the host (Figure 5A,C). Upon disruption of gut microbiota by antibiotic treatment, feeding of the VB- diet induced rapid elevation in urinary GA excretion after 2 days of feeding, and accumulation of GA in plasma was also higher than those of native gut microbiota mice after 3 and 5 days of feeding (Figure 5B,D). The magnitude of the elevation in urinary GA was higher for antibiotic-treated mice than for control mice. In control mice, urinary GA excretion increased after 7 days of VB- diet feeding. Urinary VB2 excretion was more rapidly affected by VB- diet feeding. These results suggest that urinary VB2 may reflect dietary VB2 intake and urinary GA may reflect VB2 deficiency in host liver tissues. Moreover, in mice, VB2 produced and supplied from the gut microbiota has a significant role in the VB2 status of the host after short-term depletion in dietary VB2.

## 4. Discussion

**Figure 6:**
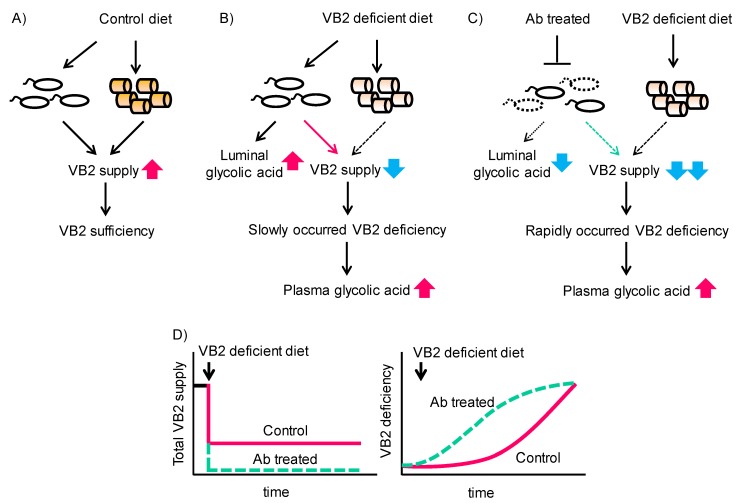
Interaction of dietary VB2, the gut microbiota, and glycolate accumulation in gut microbiota and the mouse host. (**A**) Control diet feeding with normal gut microbiota sustains a sufficient VB2 supply from both sources and preserves VB2 sufficiency. (**B**) VB2- diet feeding with normal gut microbiota sustains a VB2 supply from the gut microbiota only in the short term and before VB2 deficiency gradually develops. (**C**) Feeding of a VB2- diet in the presence of disrupted gut microbiota due to antibiotic treatment does not sustain adequate VB2 supplies and rapidly induces VB2 deficiency. (**D**) Time course of total VB2 supply (**left**) and VB2 deficiency (**right**) in mice fed the VB2- diet with or without antibiotic treatment.

Vitamins act as coenzymes for various enzymes. In this study, we found that VB2 deficiency increases GA concentration both in the gut microbiota and the host. This accumulation of GA in the host occurred concomitantly with decreased activity of GO in the liver. In this context, luminal bacteria act as a supplier of VB2, and therefore, microbial dysbiosis may accelerate vitamin B_2_ deficiency when dietary VB2 is depleted (Figure 6).

The enzyme GO, also known as (L-2-) hydroxy-acid oxidase, which metabolizes GA to glyoxylate, is widely conserved in intestinal bacteria and mammals [33,34,35,36,37,38]. VB- diet feeding induced changes in the abundances of specific phylum, such as *Firmicutes* and *Bacteroidetes*; however, unlike the change in GA concentration, no consistent changes were observed between the 2-week and 4-week treatments (Supplemental Figure S5). These results suggest that GA production in the intestinal microbiota may be caused by metabolic changes of those bacteria instead of those compositional changes by VB- diet feeding. We found that Ab-treatment reduced luminal and cecum GA concentrations in mice fed both the control or VB- diet (Figure 2B,C), whereas the plasma GA concentration was affected only by vitamin B deficiency and not by the Ab-treatment (Figure 2D). These results indicated that luminal GA may be produced by intestinal bacteria and supply to the cecum. On the other hand, elevation of plasma GA may sorely depend on the production and supply of GA from the host itself. Indeed, an increase in urinary excretion of GA has been reported in knock-out mice lacking *Hao1*, which encodes GO [31]. In the present study, VB2 supplementation of the VB- diet significantly inhibited increases in GA concentration, and when a VB2- diet was administered, significant increases were observed in plasma GA concentrations. Because GO is primarily expressed in the liver in mice [31], this organ is thought to be the primary site for production of GA from glyoxylic acid. However, the effect of increases in plasma GA or in other organs in the host is unclear because GO knockout mice do not show a specific phenotype [31]. 

The production of GA was shown to increase in rats fed a diet deficient in VB6, a coenzyme of AGT1 [39,40,41]. However, even in mice fed a VB6-deficient diet in our study, no increase in GA concentration was observed, and VB6 supplementation of the VB- diet did not affect GA concentration. These results suggest that the glyoxylic acid and GA metabolic pathways may differ between rats and mice. Indeed, the pharmacokinetics of ethylene glycol, which is involved in the glyoxylic acid metabolism pathway, do differ between rats and mice [42].

VB2 is produced by several bacteria, including *Lactobacillus*, in the gut [43] and hosts express the VB2 transporter in the distal gut [44,45,46,47,48,49]. However, only estimates of VB2 that could be produced and supplied from the gut microbiota to the host are available [50]. In that study, conducted by Magnusdottir et al., only 2.8% of dietary reference intake appeared to be supplied from gut microbiota in humans. Here, we found that disruption of the gut microbiota by antibiotic treatment of mice induced more rapid elevations in plasma GA concentration upon feeding of the VB- diet. Moreover, the gut microbiota delayed elevations in urinary GA excretion from 2 days to 7 days. These results suggest that the supply of VB2 from enteric bacteria has a significant role in host VB2 homeostasis, especially when dietary intake of vitamin B_2_ was insufficient. The time course of changes in urinary GA concentration at the time that vitamin B-deficient food is consumed is consistent with our finding that increases in GA concentration occurred later in the control group than the group that received Ab. 

Because VB2 deficiency is a risk factor for various diseases and is involved in the activation of other vitamins, proper assessment of the nutritional status for VB2 is important. Urinary VB2 (riboflavin) concentration and the erythrocyte glutathione reductase activation factor (EGRAC) are currently used as biomarkers to reflect the nutritional status of VB2 [51]. In experiments with VB- diet feeding and antibiotic treatment, the concentration of VB2 in urine decreased within 2 days of VB- diet administration in the control group to reflect VB2 intake. On the other hand, the urinary GA concentration increased 7 days after beginning administration of the VB- diet. Thus, changes in urinary GA concentration occurred more slowly than urinary VB2 levels. Changes in GA concentration may reflect VB2 sufficiency in the body because it involves enzyme activity as well as EGRAC. Evaluation of GA accumulation is simple since it increases, rather than decreases with VB2 deficiency, and in contrast to EGRAC, urinary GA levels can be easily measured. Therefore, information concerning dietary VB2 intake and the status of VB2 sufficiency/deficiency in the host can be obtained by measuring both urinary vitamins and GA as dual biomarkers of VB2 nutritional status. In addition, the ability of enterobacteria to supply VB2 can be evaluated.

## 5. Conclusions

The present study revealed that VB2 deficiency increases GA concentration, and that enterobacteria can compensate for VB2 deficiency in the host when the diet is deficient in VB2.

## Supplementary Materials

The following are available online at https://www.mdpi.com/2072-6643/12/3/736/s1, Figure S1: Key members of metabolites (in the square), enzyme (grey), and B-vitamins (yellow) in one-carbon metabolism. Figure S2: The list of metabolites identified in plasma from a metabolite list provided by HMT. Figure S3: Antibiotic treatment reduced the size of gut microbiota in mice with or without VB- diet feeding. Figure S4: VB2 intake in Experiment 4 and 5. Figure S5: Effect of VB- diet feeding on the amount of gut microbiota in mice.
