# Supplementary material for: Glycolate is a Novel Marker of Vitamin B2 Deficiency Involved in Gut Microbe Metabolism in Mice
Source: Nutrients. 2020 Mar 11;12(3):736. doi: 10.3390/nu12030736 (PMC7146322; doi:10.3390/nu12030736)
Supplement: Supplementary file 1 [file nutrients-12-00736-s001.zip › Supplemental Figure 1.pdf]

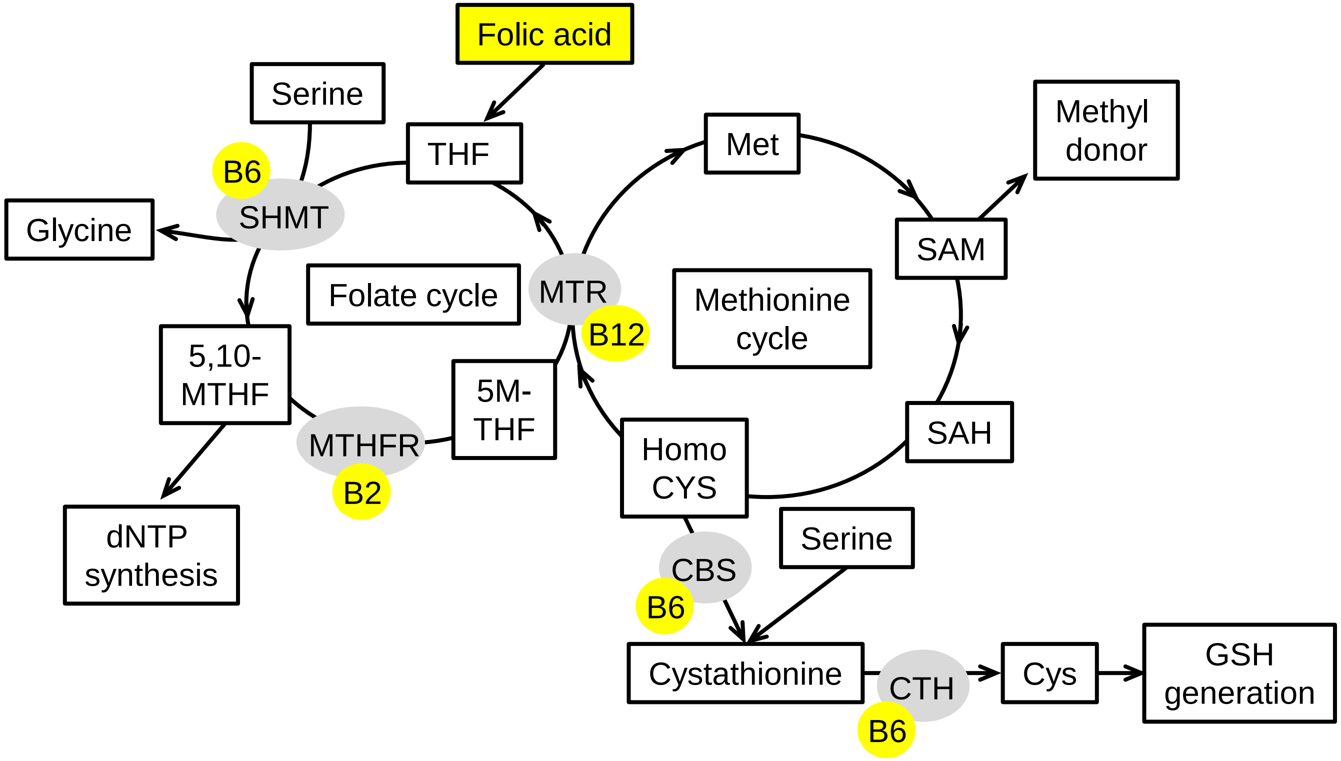

Supplemental Figure 1

Supporting Information Figure S1. Key members of metabolites (in the square), enzyme (grey), and B-vitamins (yellow) in one-carbon metabolism.

Abbreviations: 5, 10-MTHF, 5,10-methylenetetrahydrofolate; 5M-THF, 5-methyltetrahydrofolate; CBS, cystathionine β-synthase; CTH, cystathionine γ-lyase; Cys, Cysteine; Met, Methionine; MTHFR, 5,10-methylenetetrahydrofolate reductase; MTR, methionine synthase; SAM, S-adenosylmethionine; SAH, S-adenosylhomocysteine; SHMT, serine hydroxymethyltransferase; THF, tetrahydrofolate;
