# Supplementary material for: Glycolate is a Novel Marker of Vitamin B2 Deficiency Involved in Gut Microbe Metabolism in Mice
Source: Nutrients. 2020 Mar 11;12(3):736. doi: 10.3390/nu12030736 (PMC7146322; doi:10.3390/nu12030736)
Supplement: Supplementary file 1 [file nutrients-12-00736-s001.zip › Supplemental Figure 2.pdf]

Compound Name	C2w	VB-2w	C4w	VB-4w
Glycolic acid	12.6	30.4	10.0	35.3
Lactic acid	2142.8	2415.4	2119.6	1963.4
3-Hydroxybutyric acid	65.1	75.4	28.9	57.7
2-Hydroxybutyric acid	3.9	4.5	3.1	3.3
Fumaric acid	4.5	4.3	2.3	3.6
2-Oxoisovaleric acid	1.1	1.2	1.0	0.9
2-Phosphoglyceric acid	0.4	0.3	0.2	0.3
Citric acid	51.5	63.4	36.5	43.0
Isocitric acid	2.2	1.7	1.0	1.3
Gluconic acid	2.8	2.6	1.5	2.7
Ribose 5-phosphate	0.3	0.2	0.1	0.2
Glucose 6-phosphate	1.6	0.2	0.1	0.4
Fructose 6-phosphate	0.6	0.2	0.1	0.3
Glucose 1-phosphate	0.3	0.0	0.1	0.2
6-Phosphogluconic acid	0.1	0.1	0.0	0.1
UMP	0.1	0.1	0.1	0.9
cAMP	0.0	0.0	0.0	0.0
IMP	0.1	0.1	0.1	1.5
GMP	0.1	0.2	0.1	1.2
ADP	0.2	0.5	0.1	1.9
ATP	0.1	0.4	0.1	0.4
GTP	0.0	0.1	0.0	0.1
Succinic acid	50.7	59.4	28.7	42.8
Malic acid	21.0	22.3	10.7	18.3
Glycerol 3-phosphate	5.4	2.9	2.1	12.8
cis-Aconitic acid	2.1	2.2	1.3	1.5
3-Phosphoglyceric acid	0.9	0.8	0.5	1.4
Sedoneptulose 7-phosphate	0.4	0.1	0.1	0.3
AMP	0.4	0.6	0.3	9.4
GDP	0.1	0.1	0.0	0.1
Glyoxylic acid	2.7	2.2	1.9	3.1
Pyruvic acid	39.5	39.7	42.1	29.7
2-Oxoglutaric acid	11.3	10.9	8.8	7.6
Ribulose 5-phosphate	0.8	0.4	0.2	0.9
Glycine	221.4	298.4	206.8	398.2
Alanine	674.1	538.8	682.1	695.7
N,N-Dimethylglycine	5.0	7.8	3.9	7.6
Choline	31.5	26.9	26.6	76.4
Serine	155.2	171.6	176.5	215.8
Uracil	208.3	243.5	195.9	164.8
Creatinine	7.4	9.0	6.7	7.1
Proline	106.9	144.5	185.4	204.7

Compound Name	C2w	VB-2w	C4w	VB-4w
Valine	256.4	283.8	283.2	292.9
Betaine	92.0	99.7	103.0	98.7
Threonine	246.2	285.4	238.8	319.9
Betaine aldehyde_+H2O	0.4	0.3	0.4	0.3
Cysteine	1.9	1.8	1.9	2.0
Thymine	150.7	164.8	131.9	127.5
Hydroxyproline	8.1	10.9	8.3	8.1
Creatine	89.8	103.1	100.6	333.4
Isoleucine	103.2	125.2	111.0	114.9
Leucine	157.3	193.1	167.4	188.1
Ornithine	31.4	33.4	42.7	39.8
Aspartic acid	63.6	104.1	75.4	94.2
Adenine	9.7	6.4	6.1	68.9
Hypoxanthine	1.9	1.1	0.6	15.4
Anthranilic acid	0.5	0.6	0.5	0.8
Glutamine	834.6	1042.7	866.2	929.1
Lysine	519.9	638.1	577.7	489.0
Glutamic acid	50.5	25.5	36.7	330.9
Methionine	43.1	42.6	42.9	41.8
Histidine	74.9	79.2	87.8	80.5
Phenylalanine	60.4	72.7	77.0	65.4
Arginine	82.3	96.1	98.5	90.3
Citrulline	55.6	75.4	66.2	66.4
Tyrosine	81.3	101.8	106.1	87.1
Trptophan	88.4	95.4	94.7	76.1
Carnosine	1.3	1.2	1.2	0.8
Thymidine	5.6	6.3	5.8	5.4
Cytidine	2.9	3.1	2.7	4.1
Uridine	29.4	34.3	25.0	35.9
Adenosine	0.3	0.4	0.5	4.2
Inosine	2.4	0.5	1.4	25.4
Guanosine	0.3	0.0	0.1	1.9
Glutathione(GSSG)	45.9	28.0	12.0	76.7
Glutathione(GSH)	13.0	7.2	3.0	18.7
S-Adenosylmethionine	0.3	0.2	0.2	0.7

Supplemental Figure 2

Supporting Information Figure S2. The list of metabolites identified in plasma from a metabolite list provided by HMT. Relative concentration of metabolites. Values are mean of the group, and color scale showed maximum in red and minimum is green and 50 percentile is yellow.
