# Supplementary material for: Glycolate is a Novel Marker of Vitamin B2 Deficiency Involved in Gut Microbe Metabolism in Mice
Source: Nutrients. 2020 Mar 11;12(3):736. doi: 10.3390/nu12030736 (PMC7146322; doi:10.3390/nu12030736)
Supplement: Supplementary file 1 [file nutrients-12-00736-s001.zip › Supplemental Figure 3.pdf]

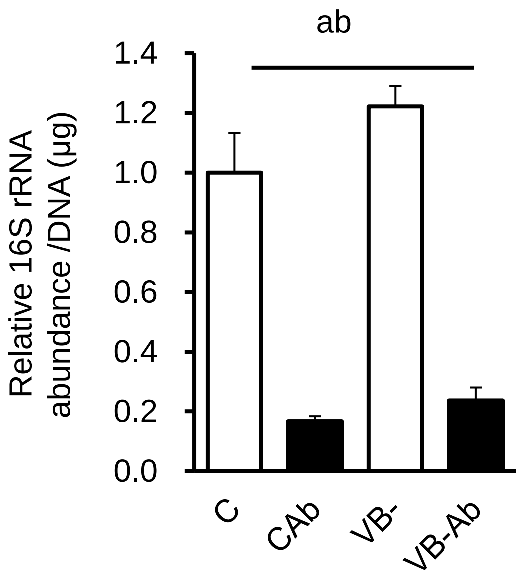

Supplemental Figure 3

Supporting Information Figure S3. Antibiotic treatment reduced the size of gut microbiota in mice with or without VB- diet feeding

A) Relative abundance of bacterial 16S rRNA/DNA in mice fed control diet or VB- diet for 4 weeks. Two-way ANOVA was used to determine the effect of diet (d), antibiotics (ab), and the interaction (i) between the two; $P < 0.05$.
