# Supplementary material for: Glycolate is a Novel Marker of Vitamin B2 Deficiency Involved in Gut Microbe Metabolism in Mice
Source: Nutrients. 2020 Mar 11;12(3):736. doi: 10.3390/nu12030736 (PMC7146322; doi:10.3390/nu12030736)
Supplement: Supplementary file 1 [file nutrients-12-00736-s001.zip › Supplemental Figure 4.pdf]

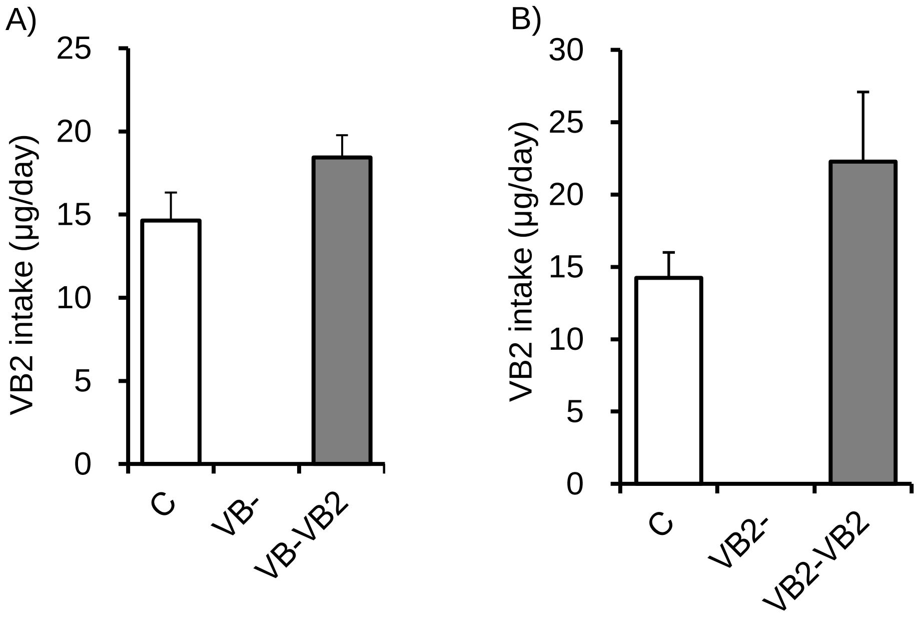

Supplemental Figure 4

Supporting Information Figure S4. VB2 intake in Experiment 4 and 5
(A, B) Average daily VB2 intake in experiment 4 (A), n=3-4 and (B) 5, n=4-5.
