# Supplementary material for: Glycolate is a Novel Marker of Vitamin B2 Deficiency Involved in Gut Microbe Metabolism in Mice
Source: Nutrients. 2020 Mar 11;12(3):736. doi: 10.3390/nu12030736 (PMC7146322; doi:10.3390/nu12030736)
Supplement: Supplementary file 1 [file nutrients-12-00736-s001.zip › Supplemental Figure 5.pdf]

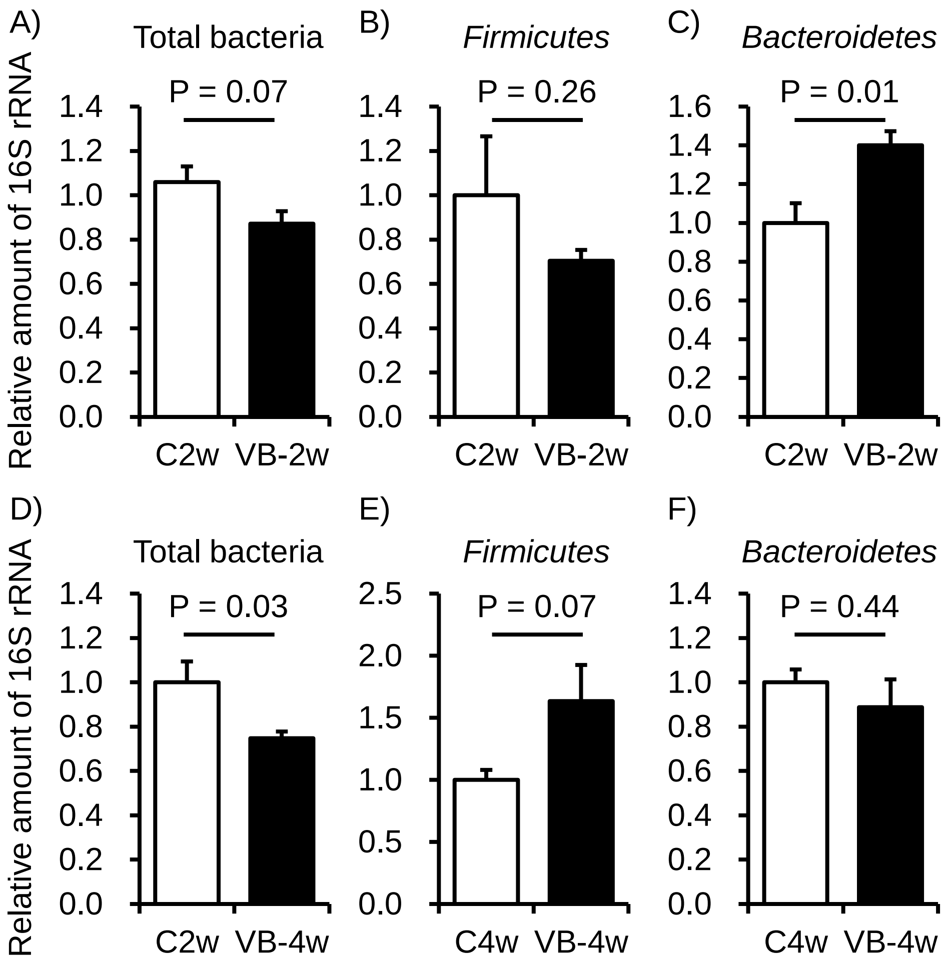

Supplemental Figure 5

Supporting Information Figure S5. Effect of VB- diet feeding on the amount of gut microbiota in mice.

Relative abundance of bacterial 16S rRNA/DNA (A and D) or specific bacterial phylum (B, C, E, F) in mice fed control diet or VB- diet for 2 or 4 weeks. P-values were caluculated by t-test.
